# IDO1 Deficiency Does Not Affect Disease in Mouse Models of Systemic Juvenile Idiopathic Arthritis and Secondary Hemophagocytic Lymphohistiocytosis

**DOI:** 10.1371/journal.pone.0150075

**Published:** 2016-02-25

**Authors:** Karen Put, Ellen Brisse, Anneleen Avau, Maya Imbrechts, Tania Mitera, Rik Janssens, Paul Proost, Francesca Fallarino, Carine H. Wouters, Patrick Matthys

**Affiliations:** 1 Laboratory of Immunobiology, Rega-Institute for Medical Research, Department of Immunology and Microbiology, KU Leuven – University of Leuven, Leuven, Belgium; 2 Laboratory of Molecular Immunology, Rega-Institute for Medical Research, Department of Immunology and Microbiology, KU Leuven – University of Leuven, Leuven, Belgium; 3 Department of Experimental Medicine and Biochemical Sciences, University of Perugia, Perugia, Italy; 4 Laboratory of Pediatric Immunology, University Hospitals Leuven, KU Leuven – University of Leuven, Leuven, Belgium; University of Lisbon, PORTUGAL

## Abstract

**Objectives:**

Indoleamine 2,3-dioxygenase-1 (IDO1) is an immune-modulatory enzyme that catalyzes the degradation of tryptophan (Trp) to kynurenine (Kyn) and is strongly induced by interferon (IFN)-γ. We previously reported highly increased levels of IFN-γ and corresponding IDO activity in patients with hemophagocytic lymphohistiocytosis (HLH), a hyper-inflammatory syndrome. On the other hand, IFN-γ and IDO were low in patients with systemic juvenile idiopathic arthritis (sJIA), an autoinflammatory syndrome. As HLH can occur as a complication of sJIA, the opposing levels of both IFN-γ and IDO are remarkable. In animal models for sJIA and HLH, the role of IFN-γ differs from being protective to pathogenic. In this study, we aimed to unravel the role of IDO1 in the pathogenesis of sJIA and HLH.

**Methods:**

Wild-type and IDO1-knockout (IDO1-KO) mice were used in 3 models of sJIA or HLH: complete Freund’s adjuvant (CFA)-injected mice developed an sJIA-like syndrome and secondary HLH (sHLH) was evoked by either repeated injection of unmethylated CpG oligonucleotide or by primary infection with mouse cytomegalovirus (MCMV). An anti-CD3-induced cytokine release syndrome was used as a non-sJIA/HLH control model.

**Results:**

No differences were found in clinical, laboratory and hematological features of sJIA/HLH between wild-type and IDO1-KO mice. As IDO modulates the immune response via induction of regulatory T cells and inhibition of T cell proliferation, we investigated both features in a T cell-triggered cytokine release syndrome. Again, no differences were observed in serum cytokine levels, percentages of regulatory T cells, nor of proliferating or apoptotic thymocytes and lymph node cells.

**Conclusions:**

Our data demonstrate that IDO1 deficiency does not affect inflammation in sJIA, sHLH and a T cell-triggered cytokine release model. We hypothesize that other tryptophan-catabolizing enzymes like IDO2 and tryptophan 2,3-dioxygenase (TDO) might compensate for the lack of IDO1.

## Introduction

Indoleamine 2,3-dioxygenase (IDO) is an immune-modulatory enzyme catalyzing the rate-limiting step in the degradation of the essential amino acid tryptophan (Trp) to kynurenine (Kyn) [1,2]. It exerts its immune-regulating functions by several means. A local depletion of Trp increases levels of uncharged transfer RNA thereby activating the amino-acid sensitive GCN2 stress-kinase pathway leading to cell cycle arrest or anergy in T cells [3]. Amino acid deficiency might further inhibit the mammalian target of rapamycin (mTOR) pathway, leading to a translational block [4]. On the other hand, increase of Kyn and other Trp metabolites act as immunologically active ligands of the aryl hydrocarbon receptor (AhR) and can as well induce cell cycle arrest, apoptosis and favor the development of regulatory T cells (Treg) [5,6]. Next to its enzymatic function, IDO was reported to act as an intracellular signaling molecule in IDO-expressing dendritic cells [7].

The IDO pathway is involved in multiple immunological processes: first Trp degradation was described to be an innate response against infections. Later on, IDO was reported to take part in maternal tolerance, to inhibit local inflammation and autoimmunity and to suppress immune responses to cancer and infections [6]. More recently it was discovered that IDO, which was renamed IDO1, bears a paralogue, IDO2, with similar enzymatic activity. Next to IDO, the degradation of Trp can be catalyzed by another enzyme, tryptophan 2,3-dioxygenase (TDO). TDO is mainly expressed in the liver and is believed to have a homeostatic role in controlling basal Trp levels. IDO1 on the other hand is expressed in most tissues and can be induced by interferons (IFNs), in particular IFN-γ, and LPS [1,2,8].

Systemic juvenile idiopathic arthritis (sJIA) is a complex autoinflammatory syndrome in children presenting with arthritis, fever, rash and/or lymphadenopathy [9]. About 10% of sJIA patients can develop a potentially fatal complication called macrophage activation syndrome (MAS), a term that refers to excessive activation of macrophages [10]. Up to 50% of sJIA patients already present with subclinical or occult MAS as evident from increased plasma levels of soluble CD163 (sCD163) and soluble CD25 (sCD25) and the presence of hemophagocytic macrophages in their bone marrow [11,12]. MAS is classified as a secondary form of hemophagocytic lymphohistiocytosis (HLH). Primary HLH has a genetic base, while secondary or reactive HLH (sHLH) develops in the context of malignancies, infections and inflammatory disorders, in the latter case it is called MAS [13]. Nevertheless, a significant percentage of sHLH patients, including those with underlying sJIA, present with HLH-associated gene defects [14,15]. Both sJIA and sHLH are characterized by the development of a cytokine storm, with important roles for IL-1β, IL-6 and IL-18 [16,17]. In sJIA patients, it has been demonstrated that high levels of IL-6 are correlated with the occurrence of arthritis, while IL-18 is associated with the development of sHLH (MAS) in sJIA patients [18]. The role of IFN-γ in sJIA and HLH/MAS is not clear: in animal models it differs from being protective to pathogenic (reviewed in Avau and Matthys and in Brisse et al. [13,19]). In patients, we recently investigated the potential role of IFN-γ by analyzing plasma levels of IFN-γ as well as IFN-γ-induced proteins [20]. To investigate IFN-γ-induced IDO activity, the ratio of Kyn to Trp was measured in plasma of sJIA and HLH/MAS patients. Similarly to levels of IFN-γ, we detected a rather low IDO activity in sJIA and a more pronounced activity in HLH/MAS patients.

The cause of excessive inflammation as seen in sJIA and sHLH is not completely understood, but one of the hypotheses is that the disease results from a defective downregulation of an immune response to an unknown trigger [16,21]. Therefore, research into the role of inflammation-tempering pathways such as IDO1, might provide further insight into the pathogenesis. Based on our previous study where we reported a rather low IFN-γ profile in sJIA patients [20], together with the disease-limiting role of IFN-γ in an sJIA mouse model [22], we believe IFN-γ plays a protective role in sJIA. Too low levels of IFN-γ (and consequently IDO1) might therefore instigate the inflammatory response in these patients. On the other hand, IFN-γ is believed to be a pathogenic factor in most models of HLH [21]. Nevertheless, IFN-γ-induced IDO1 might still act to dampen the pathogenic effects of IFN-γ in these patients.

To verify the concept that IDO1 has immune-regulatory activities in sJIA and sHLH, we investigated the impact of an IDO1-deficient setting in 3 relevant mouse models of sJIA and sHLH. Since both diseases are characterized by a cytokine storm, the role of IDO1 was also explored in a model for T cell-triggered cytokine release syndrome. Unexpectedly, in none of the animal models, symptoms were different in IDO1-KO as compared to WT mice, suggesting IDO1 does not play a role in sJIA, sHLH or T cell-triggered disease. Although no increased levels of IDO2 and TDO were detected, we hypothesize that even basal levels of these 2 other Trp-degrading enzymes might compensate for the loss of IDO1 in these mice.

## Materials and Methods

### Mice and experimental design

Mice were all bred under specific pathogen-free conditions in the Experimental Animal Centre of Leuven University. Unless stated otherwise, mice ages 6–9 weeks were used and were age- and sex-matched for grouping within each experiment. Mice were euthanized with Nembutal (Ceva, Libourne, France). Experiments were approved by the Ethics Committee of the University of Leuven (P034/2013). BALB/c IDO1-KO mice were kindly provided by Muriel Moser (ULB, Gosselies, Belgium) and were originally described by Mellor et al. [23]. WT and IDO1-KO littermates from a heterozygous breed were used for CFA immunization and anti-CD3 treatment. The mouse model of sJIA has recently been described [24]. Briefly, CFA (Difco, Franklin Lakes, NJ, USA) with added heat-killed *Mycobacterium butyricum* (1.5 mg/ml) was emulsified in an equal volume of phosphate buffered saline (PBS). 100 μl was injected subcutaneously at the base of the tail. Mice were euthanized between 21 and 29 days after CFA injection. To compare symptoms with the original sJIA model, CFA-injected IFN-γ-KO mice, bred in our animal facility, were included. Non-injected mice were included as controls. To elicit a cytokine release syndrome, BALB/c WT and IDO1-KO littermates were injected intraperitoneally (i.p.) with 75 μg anti-CD3 (kindly provided by Louis Boon, EPIRUS Biopharmaceuticals Netherlands BV) in 100 μl PBS or PBS alone [25]. 20 μg anti-CD3 was administered to analyze cytokine production in serum from eye puncture (4h) or heart puncture (24h). Blood glucose levels were measured from tail blood at different time points with glucose test strips (One Touch Vita, Zug, Switzerland). Mice were sacrificed 24h after injection. The TLR9-triggered sHLH or MAS model has been described by Behrens et al. [26]. For these experiments mice of the C57BL/6 strain were used (IDO1-KO C57BL/6 mice were provided by Francesca Fallarino and were originally derived from Jackson Laboratory (Bar Harbor, ME, USA)). Mice were injected i.p. on days 0, 2, 4, 7, and 9 with 100 μl PBS or 50 μg unmethylated CpG1826 (IDT, Coraville, IA, USA) in 100 μl PBS to elicit MAS-like disease. As a model for sHLH, we used a mouse cytomegalovirus (MCMV)-triggered mouse model that was developed in our laboratory [27]. Hereto, BALB/c WT and IDO1-KO mice were infected with 5x10^3^ PFU salivary gland-derived MCMV (Smith strain) by i.p. injection at 5 weeks of age to evoke an sHLH syndrome. Mice were sacrificed at day 5 post-infection.

### Blood analysis and flow cytometry

Heparin-anti-coagulated blood samples were obtained by heart puncture (LEO Pharma, Ballerup, Denmark). A complete blood cell analysis was performed with a Cell-Dyn 3700 hematology analyzer (Abbott Diagnostics, Abbott Park, IL, USA). For flow cytometry, single cell suspensions from spleen, inguinal lymph nodes or thymus were incubated with anti-CD16/anti-CD32 antibodies (Miltenyi Biotec, Bergisch Gladbach, Germany) and stained with the following monoclonal antibodies: FITC-labeled CD3e (145-2C11); PE-labeled CD122 (5H4), CD4 (L3T4), CD25 (PC61.5) and KI67 (B56); APC-labeled, CD49b (DX5), CD8 (53-6.7) and FOXP3 (FJK-16s); PerCP-Cy5.5-labeled CD4 (RM4-5) (all from eBioscience, San Diego, CA, USA; except for KI67 from BD Biosciences, San Jose, CA, USA). Propidium iodide (PI) was used to exclude dead cells. Staining for active caspase 3 (Casp3) was performed using a Casp3 inhibitor conjugated to FITC, according to the manufacturer’s protocol (Abcam, Cambridge, UK). Intracellular staining for KI67 and forkhead box P3 (FOXP3) were performed with the BD cytofix/cytoperm kit (BD Biosciences). Flow cytometric analysis was performed with a FACSCalibur flow cytometer (BD Biosciences) using FlowLogic analysis software (Inivai Technologies, Mentone Victoria, Australia).

### qPCR, ELISA, Western Blot

Total RNA was extracted using the PureLink RNA Mini kit (Invitrogen, Carlsbad, CA, USA). Complementary DNA was obtained using Superscript II reverse transcriptase and random primers (Invitrogen). Real-time qPCR was performed by using TaqMan FAST PCR master mix (Applied Biosystems, Foster City, CA, USA) and primer/probe pairs from Integrated DNA Technologies (IDT, Coralville, IA, USA, Gene Expression Assays: *Ido1*, Mm.PT.58.29540170; *Ido2*, Mm.PT.58.41795494; *Tdo2*, Mm.PT.58.13890594). Expression levels were normalized to the expression housekeeping genes *Gadph* (Mm.PT.39a.1) and/or *Gusb* (Mm.PT.39a.22214848) by using a geometric mean normalization method as previously described [28]. Serum cytokines were measured with a ProcartaPlex multiplex immunoassay (eBioscience). sCD25 levels were determined according to the manufacturer’s instruction (Duoset, R&D Systems, Minneapolis, MS, USA) and a sandwich ELISA detecting the heavy chain of ferritin was kindly offered by Dr. Santambrogio [29]. Plasma concentrations of aspartate transaminase (AST) and alanine transaminase (ALT) were measured spectrophotometrically using a UV-kinetic method according to the manufacturer’s instructions (ALT (SGPT) Reagent Set, AST (SGOT) Reagent Set, Teco Diagnostics, Anaheim, CA, USA). Liver and spleen tissue was lysed in PBS with 0.1% Triton (Panreac AppliChem, Darmstadt, Germany) supplemented with proteinase inhibitor (Roche, Basel, Switzerland) using a tissue homogenizer (Precellys 24, Bertin Technologies, Montigny-le-Bretonneux, France). Samples were centrifuged (20 min; 12000g, 4°C) and supernatant was used for further analysis. Protein concentration was quantified using a bicinchoninic acid (BCA, Bio-Rad Laboratories, Hercules, CA, USA) assay and samples were subjected to SDS-PAGE. Western blot analysis of IDO2 and TDO2 was performed as described [30].

### Statistical analysis

Data were analyzed by non-parametric Mann-Whitney U test for comparison of 2 groups or by Kruskal-Wallis test, followed by Dunn’s multiple comparison test for comparison of 3 or more groups. P-values < 0.05 were considered significant. GraphPad Prism software version 6.00 was used.

## Results

### IDO1-deficient mice develop a systemic inflammation comparable with wild-type mice upon challenge with CFA

**Fig 1:**
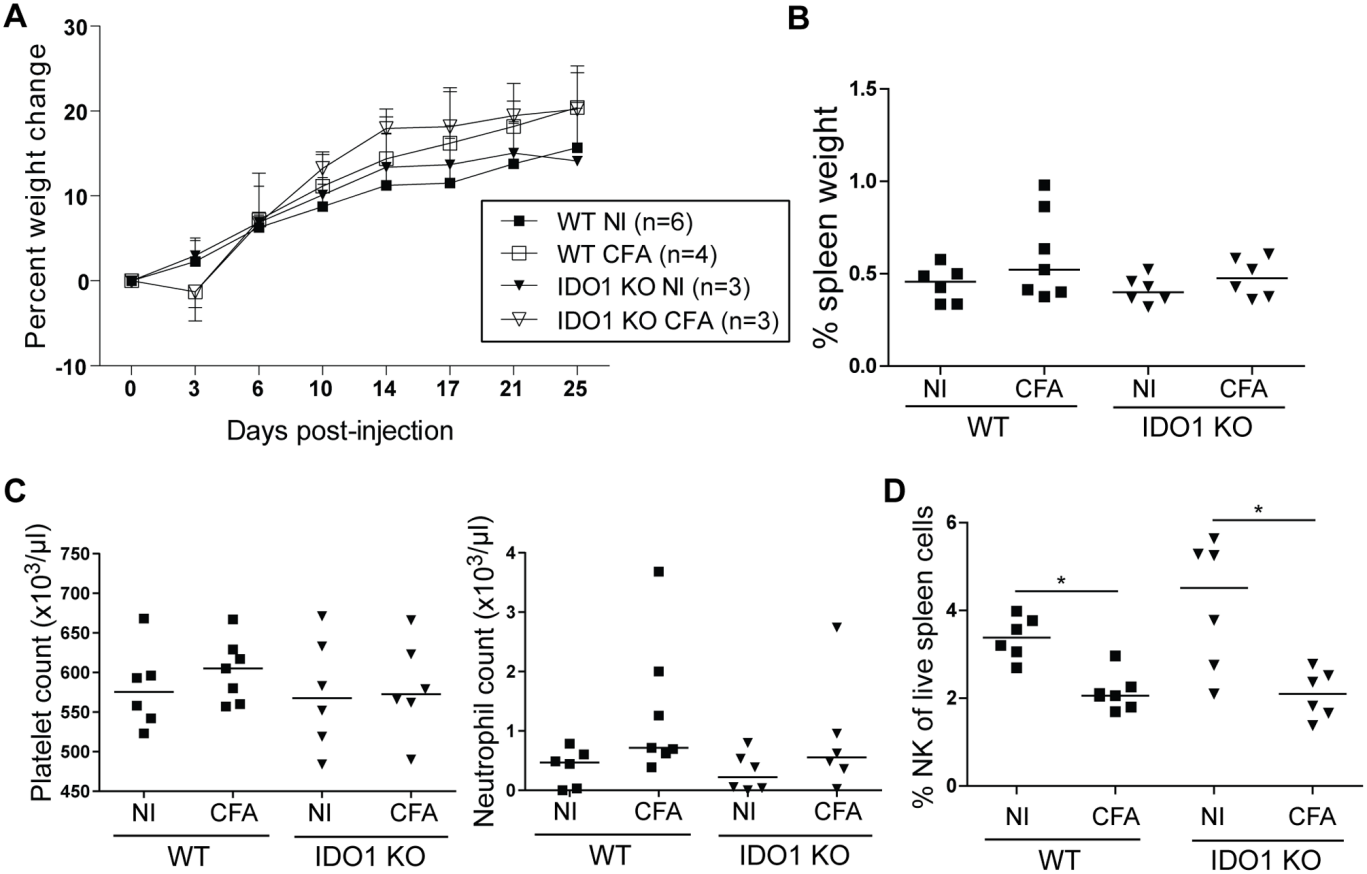
CFA injection in BALB/c WT and IDO1-KO mice. BALB/c WT and IDO1-KO mice were injected with CFA at day 0. Mice were sacrificed between day 21 and day 29 after immunization. **A.** Average ± SD percent body weight change. **B.** Spleen weight as a percentage of total body weight. **C.** Blood platelet and neutrophil counts as measured by hematology analyzer. **D.** Percentage of live CD3^-^ CD49b^+^ CD122^+^ natural killer cells of total live spleen cells. B-D: Symbols represent individual mice with the group median. Representative images of two independent experiments. * p<0.05; Kruskal-Wallis with Dunn’s post test. NI, noninjected; CFA, complete Freund’s adjuvant; NK, natural killer.

**Fig 2:**
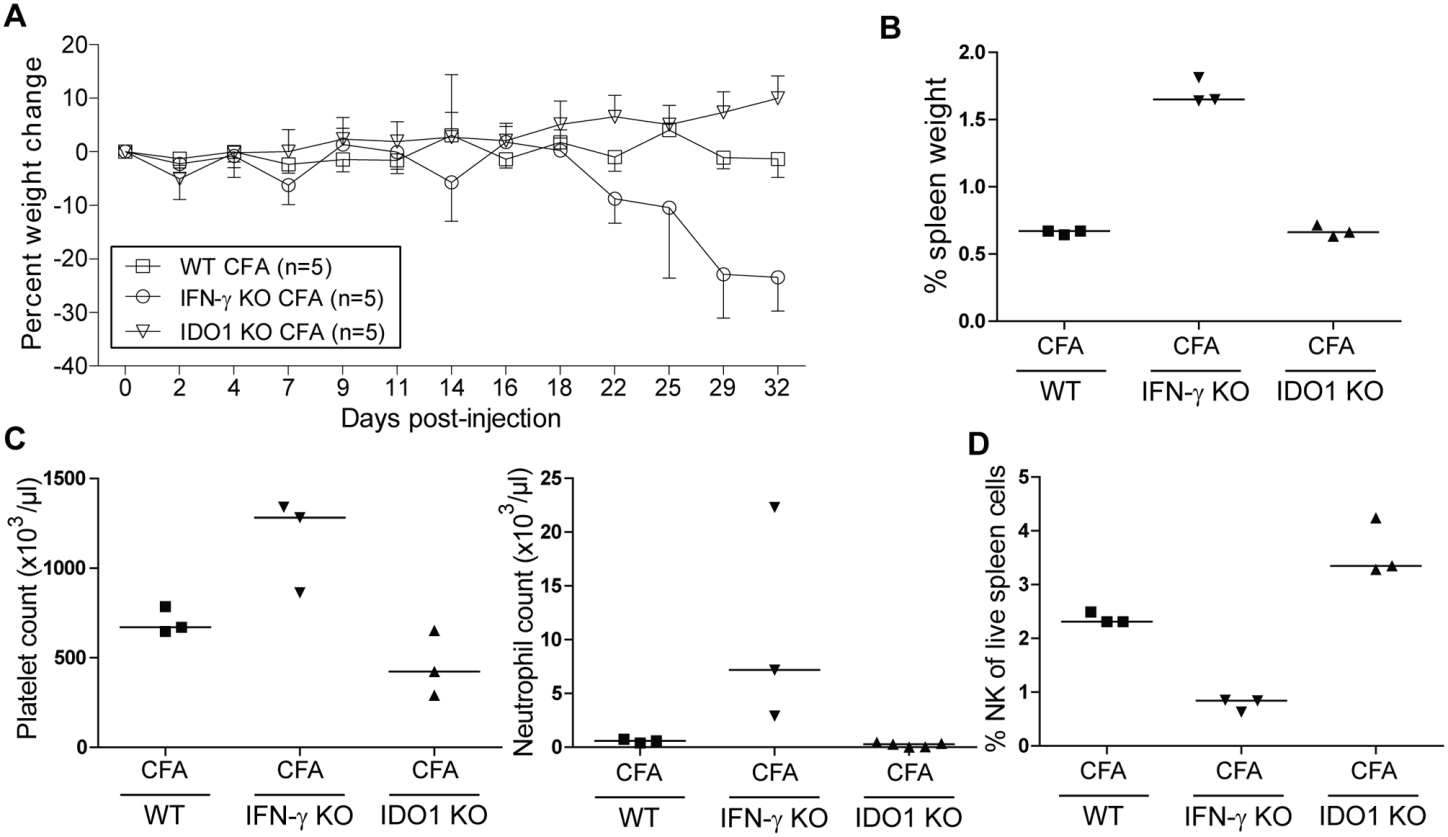
Comparison between IDO1-KO and IFN-γ-KO mice after CFA injection. CFA was injected into BALB/c WT, IDO1-KO and IFN-γ-KO mice at day 0. Mice were sacrificed at day 21 after immunization. **A**. Average ± SD percent body weight change. **B.** Spleen weight as a percentage of total body weight. **C.** Blood platelet and neutrophil counts as measured by hematology analyzer. **D.** Percentage of live CD3^-^ CD49b^+^ CD122^+^ natural killer cells of total live spleen cells. B-D: Symbols represent individual mice with corresponding group median. Data from one experiment. NI, noninjected; CFA, complete Freund’s adjuvant; NK, natural killer.

To investigate the role of IDO1 in a mouse model of sJIA, we injected complete Freund’s adjuvant (CFA) in WT and IDO1-KO BALB/c mice and compared their disease courses with IFN-γ-KO mice, in which CFA-elicited symptoms are reminiscent of sJIA [22]. In contrast to IFN-γ-KO mice, WT and IDO1-KO mice did not show wasting following immunization with CFA (Figs 1A and 2A). In both CFA-injected WT and IDO1-KO mice, spleen weight did not increase significantly (Figs 1B and 2B). When analyzing blood counts, we observed no increase in platelet counts nor neutrophils as was seen in IFN-γ-KO mice (Figs 1C and 2C). Relative abundances of spleen NK cells were significantly decreased after injection with CFA, however, there was no difference between IDO1-KO animals and their WT counterparts (Fig 1D). Moreover, NK cell numbers were lower in CFA-injected IFN-γ-KO mice (Fig 2D). Together, we conclude that IDO1 does not play a role in the systemic inflammation elicited by immunization of mice with CFA.

### IDO1 does not play a role in a TLR9-triggered MAS mouse model

**Fig 3:**
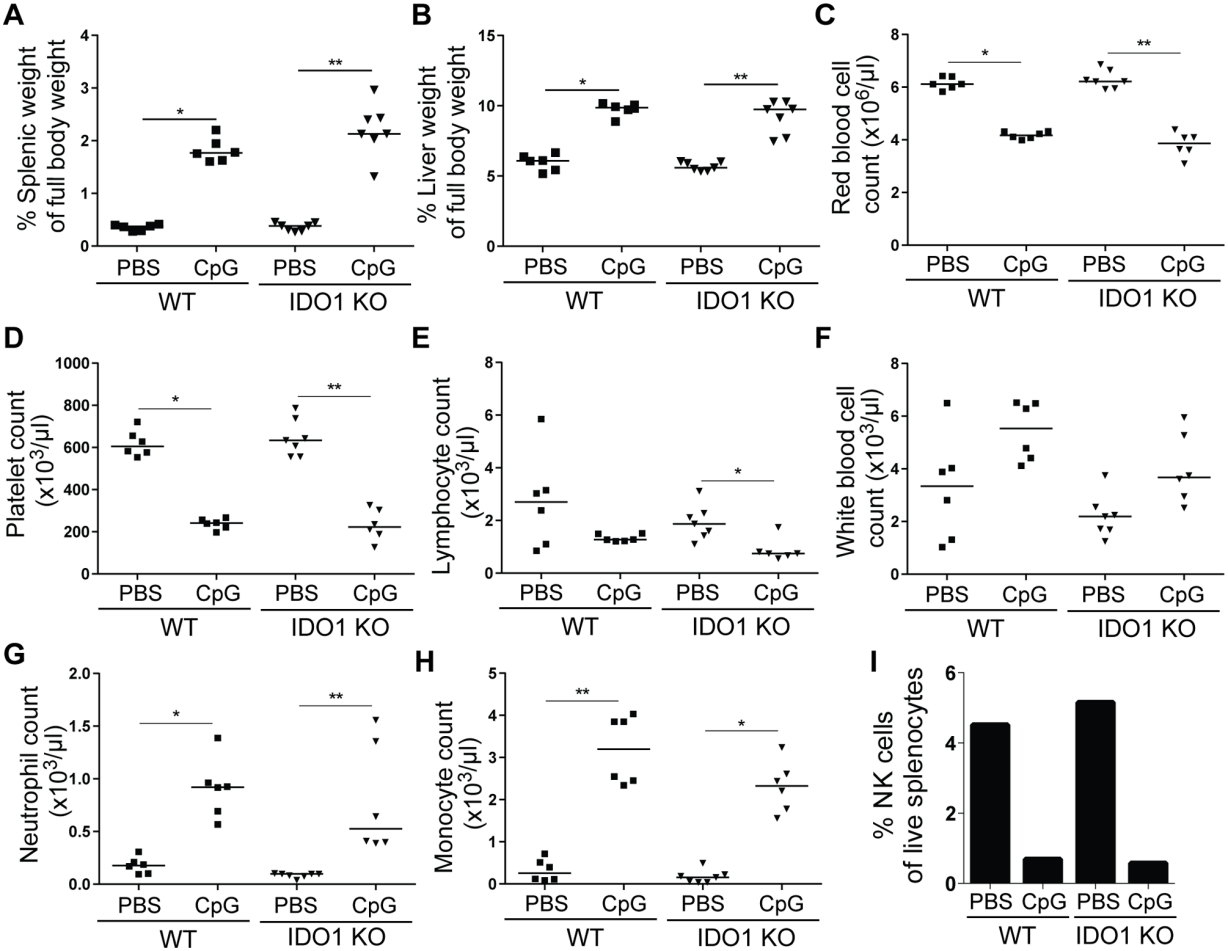
CpG-induced MAS in WT and IDO1-KO mice. C57BL/6 WT and IDO1-KO mice were repeatedly injected with PBS or CpG and were sacrificed at day 9. **A+B.** Spleen and liver weight as a percentage of total body weight. **C-H.** Red blood cell, platelet, lymphocyte, white blood cell, neutrophil, and monocyte counts in whole blood as measured by hematology analyzer. **I.** NK cell numbers were determined as CD3^-^CD49b^+^CD122^+^ cells out of total live splenocytes. A pool of splenocytes from 6–8 mice per group was analyzed. Symbols represent individual mice with the group median. Data from one experiment. * p<0.05, ** p<0.01; Kruskal-Wallis with Dunn’s post test.

We aimed to unravel the role of IDO1 in a TLR9-triggered mouse model of MAS, described by Behrens et al. [31]. In this model it was shown that IFN-γ-KO mice are protected from disease [31]. In WT mice, we confirmed the results of Behrens et al. as mice repeatedly injected with unmethylated CpG oligonucleotide showed increased spleen and liver weight (Fig 3A and 3B) and decreased numbers of circulating red blood cells, platelets and lymphocytes (Fig 3C–3E). In contrast to the original report we observed increased levels of white blood cells, neutrophils and monocytes (Fig 3F–3H). NK cell numbers in spleen were decreased after CpG injection (Fig 3I). Similar results were found in IDO1-KO mice upon CpG injection, suggesting IDO1 is neither necessary nor protective in a TLR9-triggered MAS model.

### IDO1 does not influence disease in a MCMV-infected secondary HLH mouse model

**Fig 4:**
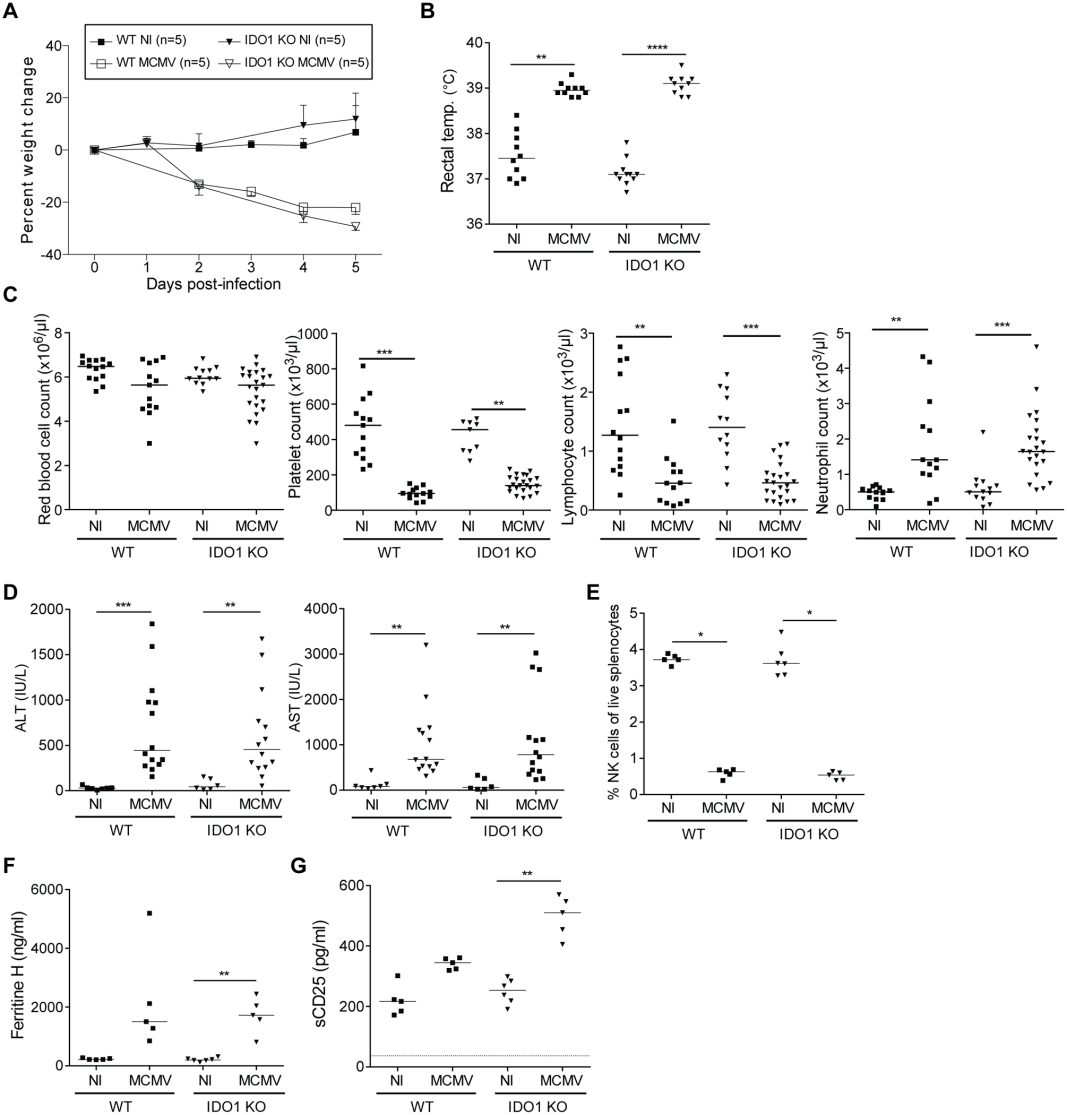
MCMV infection in the absence of IDO1. BALB/c WT and IDO1-KO mice were infected with MCMV. Mice were sacrificed at day 5 post-infection. **A.** Average percent weight change after infection. **B.** Rectal temperature at day 2 post-infection. **C.** Red blood cell, platelet, lymphocyte and neutrophil counts in whole blood as measured by hematology analyzer. **D.** Measurement of alanine and aspartate transaminase (ALT/AST) levels in plasma. **E.** Percentage of CD3^-^CD49b^+^CD122^+^ NK cells of live splenocytes. **F.** Plasma ferritin level. **G.** Plasma sCD25 level. Dotted line represents detection limit of the ELISA. Symbols represent individual mice with corresponding group median. A. Representative image from 3–4 independent experiments. B-D. Data points from 2–3 independent experiments. E-G. Data from one experiment. * p<0.05, ** p<0.01, *** p<0.001; Kruskal-Wallis with Dunn’s post test. NI, not infected; MCMV, mouse cytomegalovirus.

As IFNs play an important role in defense against viral infections, and IDO1 is specifically induced by IFNs, we investigated the response of BALB/c IDO1-KO mice to an infection with MCMV. Brisse et al. showed that MCMV infection of WT mice represents a natural model of virus-associated HLH as these mice displayed multiple features of HLH such as fever, cytopenia, hemophagocytosis, hyperferritinemia and elevated serum levels of sCD25, which were accompanied by lymphadenopathy, coagulopathy, liver dysfunction and hypercytokinemia [27]. Upon infection with MCMV, both WT and IDO1-KO mice showed severe wasting and presented with fever at day 2 post-infection (Fig 4A and 4B). At day 5 post-infection, no change in red blood cells was found, while blood platelets and lymphocytes were equally decreased in WT and IDO1-KO mice (Fig 4C). Neutrophils were highly elevated after MCMV infection, with no difference between WT and IDO1-KO mice (Fig 4C). As a measure of liver pathology, levels of liver enzymes ALT and AST were found to be increased after MCMV infection in both mouse strains (Fig 4D). Relative numbers of spleen NK cells were decreased, and plasma ferritin and sCD25 levels were increased after MCMV infection without any significant differences between WT and IDO1-KO mice (Fig 4E–4G). Although sCD25 levels were not significantly different between MCMV-infected WT and IDO1-KO mice, we did observe a trend towards higher levels of sCD25 in plasma of MCMV-infected IDO1-KO mice, which might suggest an increased T cell activation in these mice. Nevertheless, as these minor differences did not affect overall disease symptoms, we believe they are subordinate. We can conclude that similar to the other mouse models, IDO1 does not play a role in an MCMV-triggered sHLH mouse model.

### IDO1 does not affect cytokine production, number of regulatory T cells, proliferation or apoptosis in T cell-triggered inflammation

**Fig 5:**
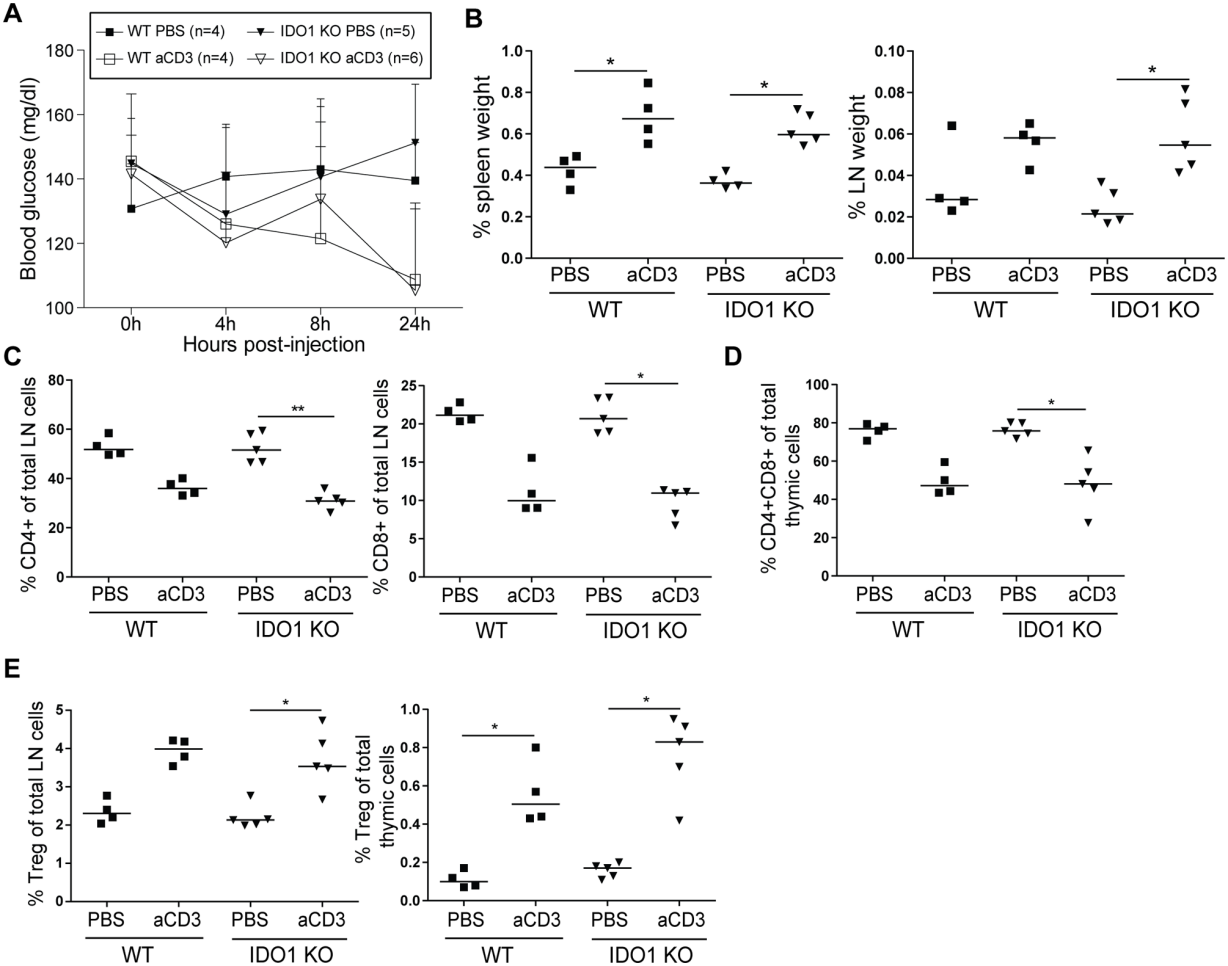
Anti-CD3-induced cytokine release syndrome in BALB/c WT and IDO1-KO mice. BALB/c WT and IDO1-KO mice were treated with a single injection of anti-CD3 or PBS, mice were monitored and sacrificed after 24h. **A.** Average ± SD blood glucose levels in mice at 0, 4, 8 and 24h post-injection. **B.** Spleen and inguinal lymph nodes weight as a percentage of total body weight. **C.** Percentage of CD4^+^ and CD8^+^ T cells of total lymph node cells. **D.** Percentage of CD4^+^CD8^+^ double positive T cells of total thymic cells. **E.** Percentages of CD4^+^CD25^+^FOXP3^+^ Treg cells of total lymph node and thymic cells. B-E: Symbols represent individual mice with corresponding group median. Data from one experiment. * p<0.05, ** p<0.01; Kruskal-Wallis with Dunn’s post test. aCD3, anti-CD3; LN, lymph node, Treg, regulatory T cells.

**Fig 6:**
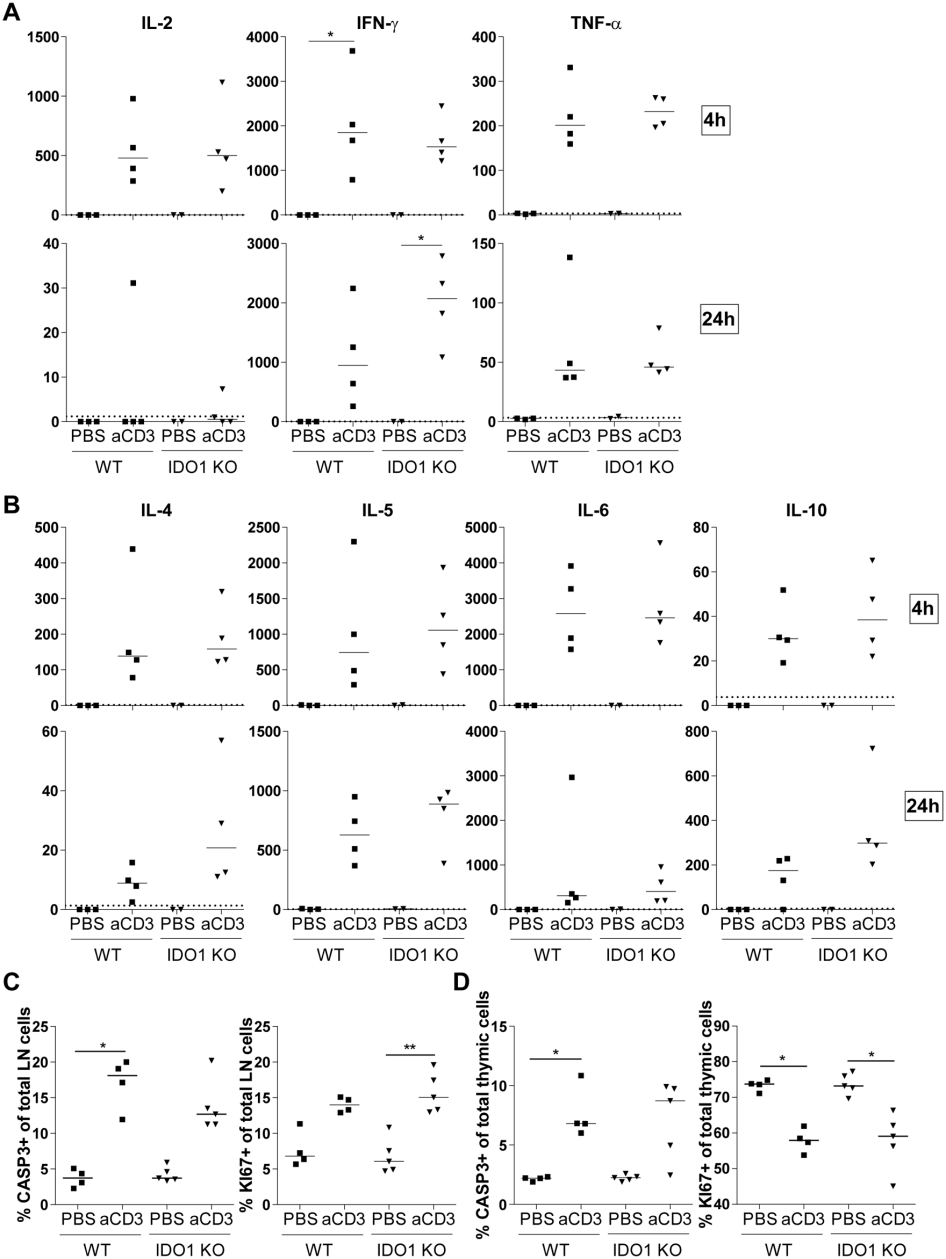
IDO1 deficiency does not affect cytokine release, proliferation and apoptosis in anti-CD3-treated mice. **A+B.**In anti-CD3- and PBS-treated BALB/c WT and IDO1-KO mice, serum Th1 cytokines (A) and Th2 and monocyte-related cytokines (B) were analyzed 4h and 24h post-injection. **C+D.** Inguinal lymph node (C) and thymic (D) cells were stained for the apoptosis marker caspase 3 (CASP3) and a proliferation marker KI67. Percentages of CASP3^+^ or KI67^+^ cells in total lymph node (C) and thymic (D) cells are shown. Symbols represent individual mice with corresponding group median. Data from one experiment. * p<0.05, ** p<0.01; Kruskal-Wallis with Dunn’s post test. aCD3, anti-CD3; LN, lymph node.

Both sJIA and especially HLH/MAS are characterized by a cytokine storm [16,17]. Furthermore, HLH/MAS patients display an excessive proliferation of T cells [10,21]. As it is known that IDO plays an important role in controlling excessive T cell proliferation, we wanted to look into the role of IDO1 in a T cell-triggered cytokine release syndrome [25]. Hereto, BALB/c WT and IDO1-KO mice were treated with anti-CD3 antibodies. Blood glucose levels were decreased after anti-CD3 injection (Fig 5A). Injection of anti-CD3 resulted in an increased weight of the spleen and inguinal lymph nodes of both WT and IDO1-KO mice (Fig 5B). Further, anti-CD3 elicited a decrease in relative abundances of CD4^+^ and CD8^+^ T cells in the inguinal lymph nodes (Fig 5C) and a decrease of immature CD4^+^CD8^+^ thymocytes, a well described feature of the anti-CD3 cytokine release syndrome in mice (Fig 5D) [25]. The percentage of FOXP3^+^ Treg cells in the inguinal lymph nodes and the thymus was increased in both WT and IDO1-KO mice after treatment with anti-CD3 (Fig 5E). Overall, no differences were observed in IDO1-KO mice as compared to their WT counterparts. Next, serum cytokine levels were analyzed 4h and 24h after anti-CD3 treatment. Anti-CD3 treatment increased serum levels of Th1 cytokines IL-2, IFN-γ and TNF-α (Fig 6A) and Th2 cytokines IL-4, IL-5 (Fig 6B) to a similar extent in both WT and IDO1-KO mice. Other, more monocyte-related cytokines IL-6 and IL-10, were similarly increased in IDO1-KO and WT mice, upon challenge with anti-CD3 (Fig 6B). Except for IL-10, levels of most cytokines decreased after 24 hours (Fig 6A and 6B). To verify whether IDO1 affects T cell proliferation and apoptosis, we stained for the proliferation marker KI67 and the apoptosis marker CASP3. In both lymph node and thymus, we found no difference in the increase of apoptosis after anti-CD3 treatment between WT and IDO1-KO mice (Fig 6C and 6D). There was an increase in proliferating lymph node cells, while the percentage of proliferating thymus cells was decreased (Fig 6C and 6D). Again no difference was found between WT and IDO1-KO animals. In conclusion, genetic ablation of IDO1 does not influence cytokine production, numbers of Treg cells, proliferation or apoptosis after anti-CD3 treatment.

### No differences in IDO2 or TDO levels between wild-type mice and IDO1-deficient mice

**Fig 7:**
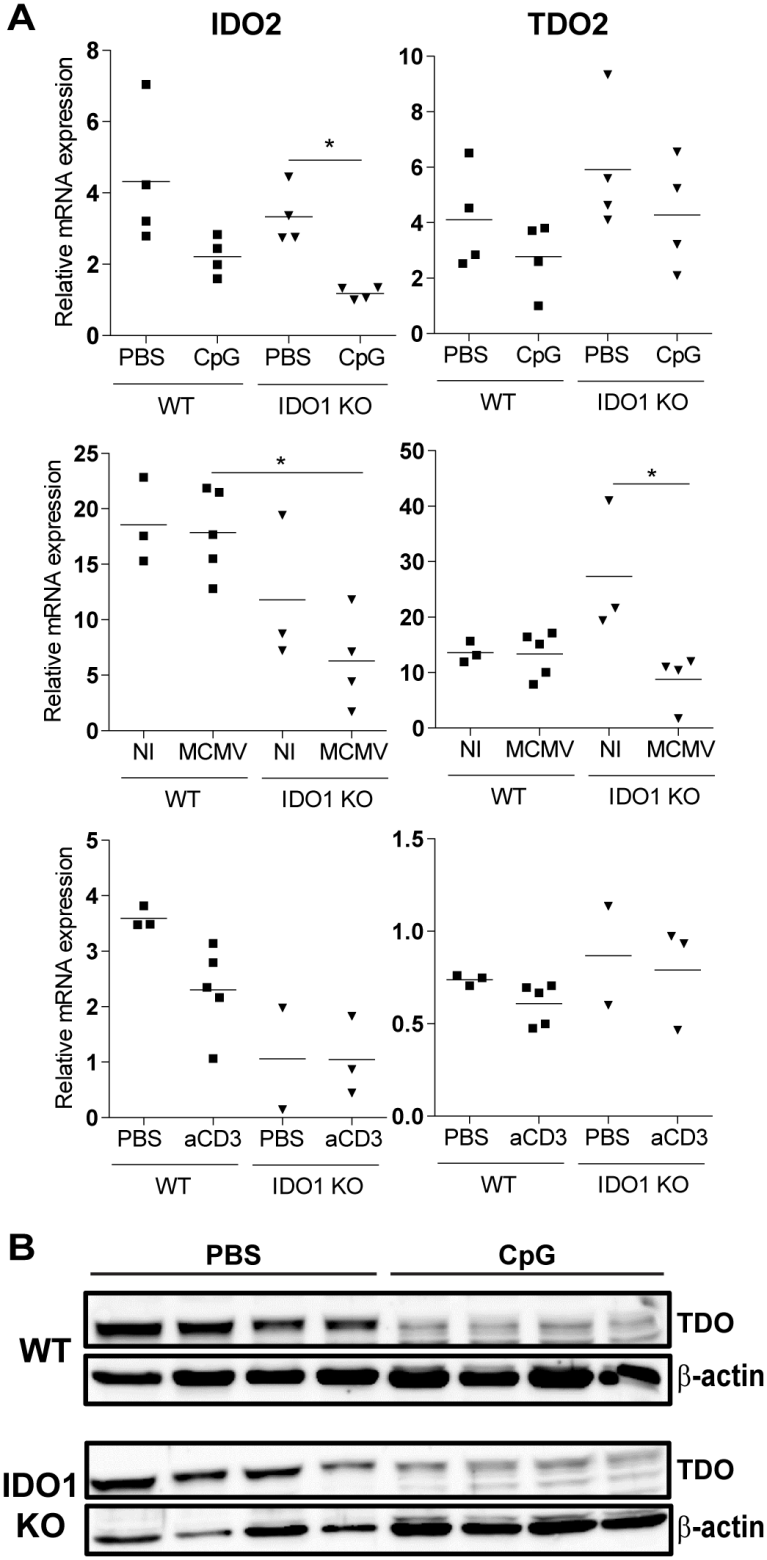
Expression of IDO2 and TDO in liver of WT and IDO1-KO mice. **A.** *Ido2* and *Tdo2* expression was assessed by qPCR in liver of CpG-, MCMV- and anti-CD3-injected mice. Expression levels were normalized to *Gapdh* (CpG model) or *Gapdh* together with *Gusb* (MCMV and anti-CD3 model). * p<0.05; Kruskal Wallis plus Dunn’s post test. NI, not infected; aCD3, anti-CD3. Data from one experiment. **B.** WT and IDO1-KO C57BL/6 mice were repeatedly injected with PBS or CpG and TDO protein expression was measured by western blot analysis in liver lysates at day 9 in 4 mice per group. Expression of β-actin was included as a loading control.

Aside from IDO1, IDO2 and TDO can catalyze degradation of Trp and might be capable of compensating the loss of IDO1 in these mice. We therefore analyzed IDO2 and TDO2 mRNA levels in liver of CpG-, MCMV- and anti-CD3-injected mice (Fig 7A). We did not observe increased expression of IDO2 or TDO2 in IDO1-KO mice. IDO2 mRNA even showed a trend towards lower levels in IDO1-deficient mice, which might potentially be linked to the genetic interaction of IDO1 and IDO2 [32]. In addition, we analyzed the protein levels of IDO2 and TDO by western blot in cell lysates of liver of WT and IDO1-KO C57BL/6 mice treated with PBS or CpG. IDO2 was not detected (data not shown). TDO was expressed by liver cells with similar levels in WT and IDO1-KO mice and CpG treatment decreased TDO expression (Fig 7B). We can conclude that the absence of IDO1 is not compensated by clear-cut increased IDO2 or TDO2 expression.

## Discussion

We previously reported a limited IFN-γ profile with low IDO activity in plasma of sJIA patients and a significant IFN-γ-profile with highly raised IDO in HLH patients [20]. IFN-γ is a potent inducer of IDO1 [6]. To investigate the immune-modulatory role of IDO1 in sJIA and sHLH, we used 3 recently described animal models for sJIA and sHLH, in which a role of IFN-γ was demonstrated by using specific neutralizing antibodies or IFN-γ gene-deficient mice [22,31].

In a CFA-injected mouse model, sJIA-like symptoms are elicited in IFN-γ-KO mice or in WT mice injected with IFN-γ neutralizing antibodies, indicating IFN-γ provides protection in the disease [22]. IFN-γ possesses both pro- and anti-inflammatory properties, with induction of IDO1 as an example of the latter [33]. Amongst others, IDO can stimulate induction of Treg cells, while it can inhibit Th17 cells. A Treg/Th17 imbalance has been suggested in sJIA [16], and might thus be linked to the particularly low IDO activity in these patients. Nevertheless, we did not find evidence for a role of IDO1 in the sJIA mouse model.

The role of IFN-γ in HLH/MAS seems different, as levels of IFN-γ and downstream proteins, including IDO, are highly increased in HLH/MAS [20,34,35]. In mouse models of primary HLH and in the CpG-triggered MAS model used in the current study, IFN-γ is pathogenic, as anti-IFN-γ antibodies could ameliorate disease and relieved most symptoms [31,36,37]. In contrast, in the MCMV-associated sHLH model described by Brisse et al., IFN-γ was protective [27]. Taken together, the data indicate that the role of IFN-γ in HLH and sJIA is complex and is most likely related to its bivalent features, i.e. its pronounced pro- and anti-inflammatory activities. As IDO has an important immune-modulatory function, one would expect an aggravation of disease in the absence of IDO. However, in both the CpG-triggered MAS model and the MCMV-triggered sHLH model, no differences were observed between WT and IDO1-KO mice. As the absence of IDO1 did not seem to influence disease in different inflammatory mouse models, we investigated IDO1 expression in WT mice infected with MCMV and treated with CpG. In white blood cells, spleen and liver, MCMV infection induced the expression of IDO1 mRNA (S1 Fig). Similar data were obtained in liver of CpG-injected mice (S1 Fig). Thus, the lack of differences in disease outcome between IDO1-KO mice and WT counterparts cannot be explained by the absence of IDO induction in these sHLH models. We additionally measured IDO1 mRNA levels in the sJIA mouse model. Although we found (not significantly) increased IDO1 mRNA in lymph nodes of CFA-injected WT animals, IDO1 levels were decreased in spleen and liver of these mice (S1 Fig). These relatively low levels of IDO1 are in line with the IDO activity we found to be low in plasma of sJIA patients. In the anti-CD3 model, IDO1 mRNA levels were not increased (S1 Fig). For the anti-CD3 cytokine release model, we can therefore not exclude that a lack of differences is simply caused by a lack of involvement of IDO1.

Although IDO is involved in inducing apoptosis, inhibiting T cell proliferation and promoting Treg differentiation, we did not find differences in these features between WT and IDO1*-*KO mice in a T cell-triggered cytokine release syndrome. Studies investigating the contribution of IDO to Treg differentiation have predominantly been performed *in vitro* or *ex vivo*, using Treg suppression assays. On the other hand, *in vivo* studies reported the abnormal differentiation of antigen-specific Treg cells after mucosal antigen challenge [6]. Treg cells of anti-CD3-challenged IDO1-KO mice might show decreased functionality in *ex vivo* suppression assays. In addition, since the anti-CD3 model does not trigger T cells in an antigen-specific matter, this might as well explain the lack of an observed difference in IDO1-KO mice.

IDO1 is believed to play an important role in acquired tolerance, nevertheless, IDO1-deficient mice do not develop spontaneous lethal autoimmune disorders. However it was shown in different ways that inhibiting IDO abrogates acquired immunity against new antigens, for example in transplanted tissues [6]. IDO has been reported to be immunosuppressive in different autoimmune models such as experimental autoimmune encephalomyelitis [38,39] and collagen-induced arthritis [40,41], as demonstrated by using pharmacological inhibitors of IDO [39,40] and by using IDO1-deficient mice [38,41]. With respect to systemic lupus erythematosus, IDO1-KO mice that were chronically exposed to apoptotic cells developed a lupus-like syndrome and pharmacological inhibition of IDO accelerated disease in the MRL-lpr mouse model for spontaneous lupus [42]. Except for the anti-CD3-induced model, all disease models in our study rely on an innate immune stimulus and all mouse models lack a specific antigenic stimulus. Since the absence of IDO1 does not influence disease in any of the inflammatory mouse models we described here, one could argue that IDO1 is important in autoimmune, rather than in autoinflammatory disease.

IDO was initially described as an antimicrobial molecule. Multiple *in vitro* studies revealed restricted replication of different intracellular pathogens, among which *Toxoplasma gondii*, *Chlamydia* species and viruses [6]. *In vivo*, Hoshi et al. reported a decreased viral replication due to increased type I IFNs in IDO1-KO mice after infection with LP-BM5 murine leukemia virus [43]. In our MCMV-associated sHLH mouse model, mice were sacrificed at day 5 post-infection, and IDO1-KO mice had similar viral titers as compared to WT mice (S2 Fig). In contrast to the acute virus infection that is described in the current study, Hoshi et al. detected differences in viral load at 8 weeks post-infection, suggesting IDO1 might play a role in the chronic phase of infection. Interestingly, when looking at the *in vivo* effect of IDO1 by using IDO1-deficient mice, Divanovic et al. reported genetic ablation of the IDO1 gene did not affect *Toxoplama gondii* infection in mice, while pharmacologic inhibition of IDO led to increased mortality and parasitic burden [44].

An explanation for the different results obtained with IDO1-KO mice as compared to those using a pharmacological IDO inhibitor, may lie in the discovery of other enzymes catalyzing the degradation of Trp and that are target of pharmacological inhibition. Before the discovery of IDO2, most studies investigating the role of IDO used a pharmacological IDO inhibitor 1-methyl-Trp (1MT), a molecule blocking both the IDO1 and IDO2 enzyme. Moreover, IDO1 and IDO2 can genetically interact, instigating a critical role for IDO2 in IDO1-mediated T cell regulation [32]. Although some studies reported IDO2 mRNA to be induced by IFN-γ as well, this could not always be confirmed [8]. Additionally, TDO, which is mainly expressed in the liver and less subjective to induction by inflammatory triggers, can also degrade Trp to Kyn [1]. Since IDO1-deficiency does not affect disease in both sJIA and sHLH mouse models, we hypothesized enzymatic activity of IDO2 and TDO might compensate for the absence of IDO1. In contrast to our previous study in patients with sJIA and HLH/MAS [20], we were not able to measure IDO activity (e.g. Kyn/Trp ratios) in plasma of mice, due to borderline detection levels of Kyn. Nevertheless, Trp levels were detectable and were slightly but not significantly decreased upon challenge with CpG, also in the absence of IDO1, suggesting compensated tryptophan degradation in IDO1-KO mice (data not shown). Even though we did not find increased expression of IDO2 or TDO mRNA nor protein levels in IDO1-KO mice after repeated CpG injection or after MCMV infection, homeostatic levels of these enzymes might already be sufficient to compensate for the loss of IDO1. Analysis of Kyn/Trp levels in these mice could clarify these assumptions and treatment with 1MT could attribute a role to both IDO1 and IDO2.

In conclusion, the absence of IDO1 does not affect disease in neither a CFA-elicited mouse model for sJIA, a TLR9-triggered MAS model, an MCMV-associated sHLH model, nor in mice with anti-CD3-elicited cytokine release syndrome. Although we could not detect increased expression of two other Trp-degrading enzymes, IDO2 and TDO, we hypothesize that even basal levels of these enzymes might compensate for the lack of IDO1 in these mice.

## Supporting Information

S1 Fig IDO1 expression in inflammatory mouse models. (PDF)

S2 Fig Viral titer in MCMV-infected mice. (PDF)
