# Supplementary material for: IDO1 Deficiency Does Not Affect Disease in Mouse Models of Systemic Juvenile Idiopathic Arthritis and Secondary Hemophagocytic Lymphohistiocytosis
Source: PLoS One. 2016 Feb 25;11(2):e0150075. doi: 10.1371/journal.pone.0150075 (PMC4767214; doi:10.1371/journal.pone.0150075)
Supplement: S1 Fig — (PDF) [file pone.0150075.s001.pdf]

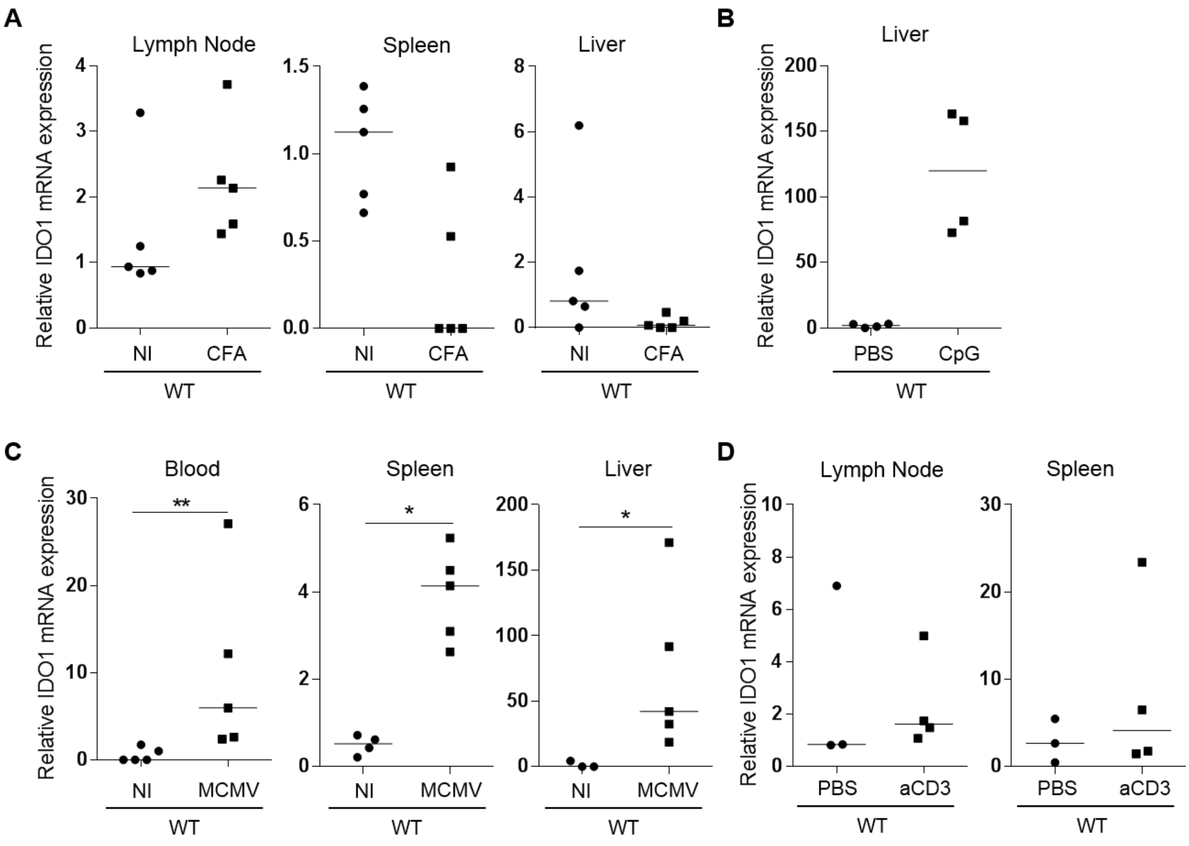

Supplementary Fig S1. IDO1 mRNA levels in inflammatory mouse models. IDO1 mRNA expression was determined in different organs in WT BALB/c mice whether or not immunized with CFA at day 21 post-immunization (A), in WT C57BL/6 mice repeatedly injected with PBS or CpG at day 9 (B), in BALB/c WT infected with MCMV on day 5 after infection (C) and in WT BALB/c mice injected with anti-CD3 at 24 hours (D). Relative mRNA levels were normalized to the expression of *Gapdh* alone (C, blood and B) or together with *Gusb* (A, C, spleen and liver, D). * $p<0.05$, ** $p<0.01$; Mann-Whitney U test. NI, not immunized or not infected; MCMV, mouse cytomegalovirus; aCD3, anti-CD3.
