# Supplementary material for: IDO1 Deficiency Does Not Affect Disease in Mouse Models of Systemic Juvenile Idiopathic Arthritis and Secondary Hemophagocytic Lymphohistiocytosis
Source: PLoS One. 2016 Feb 25;11(2):e0150075. doi: 10.1371/journal.pone.0150075 (PMC4767214; doi:10.1371/journal.pone.0150075)
Supplement: S2 Fig — (PDF) [file pone.0150075.s002.pdf]

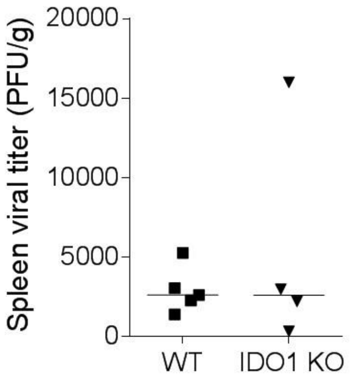

Supplementary Fig S2. Viral titer in MCMV-infected mice. The amount of infectious virus present in spleen of MCMV-infected WT and IDO1-KO mice was determined by a plaque detection assay using a tenfold titration of the supernatant of disrupted organ samples on the C127I mouse mammary gland epithelial cell line (CRL-1616, ATCC). Viral titers are depicted as number of plaque forming units (PFU) per spleen weight.
